# Genetically engineered flavonol enriched tomato fruit modulates chondrogenesis to increase bone length in growing animals

**DOI:** 10.1038/srep21668

**Published:** 2016-02-26

**Authors:** Dharmendra Choudhary, Ashutosh Pandey, Sulekha Adhikary, Naseer Ahmad, Chitra Bhatia, Sweta Bhambhani, Prabodh Kumar Trivedi, Ritu Trivedi

**Affiliations:** 1 CSIR-Central Drug Research Institute (CSIR-CDRI), Endocrinology Division, Jankipuram Extension, Sitapur Road, Lucknow-226031, INDIA; 2 CSIR-National Botanical Research Institute (CSIR-NBRI), Rana Pratap Marg, Lucknow-226 001, INDIA

## Abstract

Externally visible body and longitudinal bone growth is a result of proliferation of chondrocytes. In growth disorder, there is delay in the age associated increase in height. The present study evaluates the effect of extract from transgenic tomato fruit expressing *AtMYB12* transcription factor on bone health including longitudinal growth. Constitutive expression of *AtMYB12* in tomato led to a significantly enhanced biosynthesis of flavonoids in general and the flavonol biosynthesis in particular. Pre-pubertal ovary intact BALB/c mice received daily oral administration of vehicle and ethanolic extract of wild type (WT-TOM) and transgenic *AtMYB12*-tomato (MYB12-TOM) fruits for six weeks. Animal fed with MYB12-TOM showed no inflammation in hepatic tissues and normal sinusoidal Kupffer cell morphology. MYB12-TOM extract significantly increased tibial and femoral growth and subsequently improved the bone length as compared to vehicle and WT-TOM. Histomorphometry exhibited significantly wider distal femoral and proximal tibial growth plate, increased number and size of hypertrophic chondrocytes in MYB12-TOM which corroborated with micro-CT and expression of BMP-2 and COL-10, marker genes for hypertrophic cells. We conclude that metabolic reprogramming of tomato by AtMYB12 has the potential to improve longitudinal bone growth thus helping in achievement of greater peak bone mass during adolescence.

Both childhood and adolescence are the important periods for the growth of skeleton. During these periods, bone growth leads to increased size and strength of individual bones^1^,^2^. Children progressively accumulate bone mass, from birth to the adulthood and this accrual is referred to as the peak bone mass (PBM), ensuing lower risk of fractures or osteoporosis in later stages of life^3^. Linear growth of the appendicular skeleton is the product of a cascade of events that takes place in the cartilaginous growth center of the long bones, termed the epiphyseal growth plate^4^. The growth plate consists of three principal zones: resting, proliferative and hypertrophic. The resting zone lies next to the epiphyseal bone and contains infrequently dividing chondrocytes. However, the proliferative zone contains replicate chondrocytes arranged in columns parallel to the long axis of the bone^5^. Studies suggest that the proliferative chondrocytes located farthest from the resting zone stop replicating and enlarge to become hypertrophic chondrocytes which subsequently form bone. In addition, these cells also maintain the columnar alignment in the hypertrophic zone. The process of proliferation and hypertrophy with cartilage matrix secretion results in chondrogenesis. Simultaneously, the metaphyseal border of the growth plate is invaded with the blood vessels and bone cell precursors that remodel the newly formed cartilage into bone. This synchronized process of chondrogenesis with cartilage ossification leads to longitudinal bone growth. In humans, the process of epiphyseal fusion terminates longitudinal bone growth and the growth plate is resorbed at the time of sexual maturation^6^,^7^.

Phytoestrogens have shown to have positive effect on chondrocytes^8^,^9^. Proteoglycans (PG) consisting of low and high sulfated glycosaminoglycans (GAG) is the main components of cartilage matrix. Claassen *et al*., (2008) demonstrated that GAG synthesis and sodium [^35^S] sulfate incorporation in female bovine articular chondrocytes is affected by flavonoids, daidzein and genistein^10^. In addition, it has been reported that genistein reduces the production of cyclooxygenase (COX)-2 as a proinflammatory molecule in human chondrocytes^11^.

Flavonoids are plant secondary metabolites that act as phytoestrogens and exhibit a range of health beneficial properties including positive effect on bone health^12^,^13^. Most of the commonly consumed foods are deficient in flavonoid contents and therefore development of genetically modified plants with enhanced flavonoid content is desirable from health perspective^12^. The current study was designed, to investigate effect of genetically engineered transgenic tomatoes (MYB12-TOM) expressing a flavonol specific transcription factor from *Arabidopsis*, AtMYB12^14^. The fruits of MYB12-TOM accumulated significantly higher amount of flavonols as compared to wild type tomatoes (WT-TOM). We used extract from MYB12-TOM fruits to study their effect on peak bone mass achievement and longitudinal bone growth in pre-pubertal female mice. Our results demonstrate that growing female mice fed with extract from MYB12-TOM affected formation, quality and length of the bone. Extract from MYB12-TOM significantly increased the length by interstitial growth of the epiphyseal plate of bones by the expansion of the lacunae in the hypertrophic cells in pre-pubertal stage. Observations that expansion of the lacunae in the hypertrophic cells leads to the increased longitudinal growth is very well reported in previous studies^15^,^16^. Overall, the study signifies that metabolic engineering of plants using *AtMYB12* led to an increase in the longitudinal height of bones by supplementation to growing female mice. This growth is the resultant of endochondral proliferation of cells in growth plate region and that led to conversion of proliferating chondrocytes into hypertrophic cells and then to new bone formation.

## Results

### Analysis of flavonoid content in fruits of tomato transgenic lines

**Figure 1:**
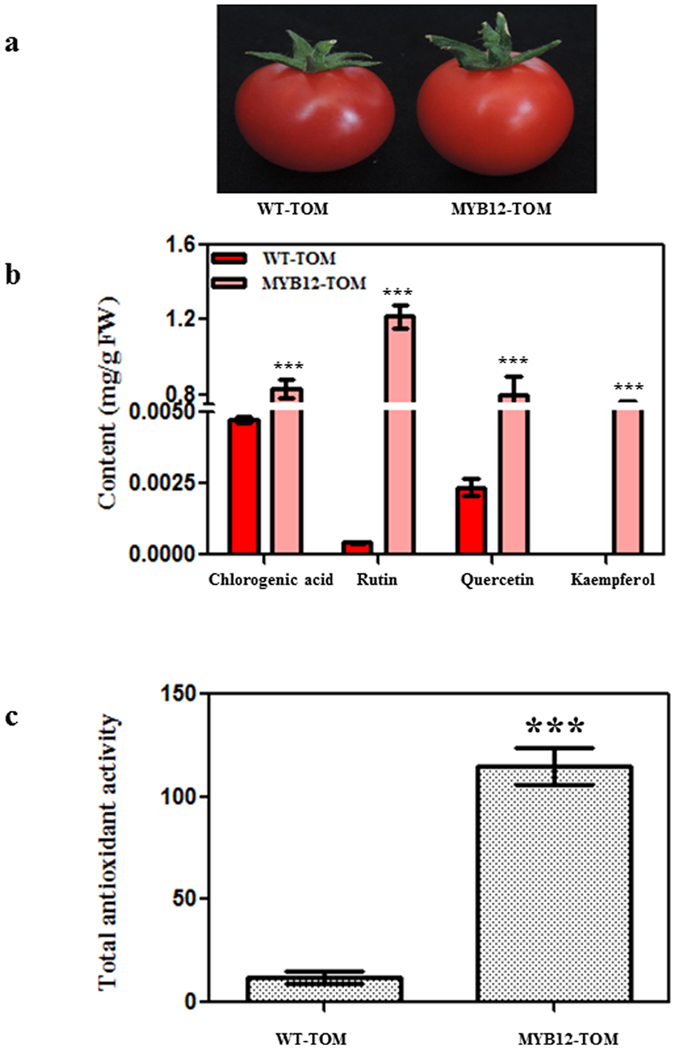
Phytochemical analysis of the methanolic extracts of fruits of *AtMYB12*-expressing tomato transgenic lines. (**a**) Mature wild type (WT-TOM) and transgenic (MYB12-TOM) tomato fruits. (**b**) Content of individual compounds was quantified by separating methanolic extracts of WT and transgenic lines using HPLC. Quantification of CGA and rutin were carried out by using non-hydrolyzed extract while quercetin and kaempferol were quantified by carried out acid-hydrolyzed methanolic extract. (**b**) Measurement of total antioxidant activity using methanolic extracts. Graph has been plotted by Trolox equivalent antioxidant capacity (TEAC). The graph shows Mean ± SD of three samples from each of the independent transgenic line. The photograph was taken by AP.

Homozygous transgenic lines expressing *AtMYB12* under control of constitutive promoter CaMV35S and WT plants were analyzed to confirm the reported modulation in flavonoid accumulation^17^. Visually, the mature ripe MYB12-TOM fruits were slightly orange as compared to being red in case of WT-TOM plants (Fig. 1a). Quantitative phytochemical analysis was carried out for estimation of important phenolics and flavonoids such as CGA and flavonols (e.g. rutin, quercetin and kaempferol) found in extract of tomato fruit using HPLC. The contents of CGA and rutin were significantly enhanced in fruits of various MYB12-TOM lines (CGA, 0.8 ± 0.07 mg g^−1 ^FW; rutin, 1 ± 0.06 mg g^−1 ^FW) as compared to the fruits of WT-TOM (CGA, 0.005 ± 0.001 mg g^−1 ^FW; rutin, 0.03 ± 0.001 mg g^−1 ^FW) (Fig. 1b). To quantify specifically aglycone of flavonoids, extracts from MYB12-TOM and WT-TOM fruits were acid-hydrolyzed and analyzed. The analysis suggested significantly enhanced levels of quercetin (up to 0.8 ± 0.1 mg g^−1 ^FW) and kaempferol (0.7 ± 0.06 mg g^−1 ^FW) in MYB12-TOM fruits as compared to WT-TOM fruits. As a reflection of enhanced polyphenol and flavonoid content, the total antioxidant capacity of MYB-TOM in terms of trolox equivalents was also enhanced more than 5 fold as compared to WT-TOM (Fig. 1c). Taken together, these results demonstrate that constitutive expression of *AtMYB12* in tomato leads to enhanced CGA, flavonol content and enhances antioxidant potential of the tomato fruit as already reported by Pandey *et al*., (2015)^17^.

### Effect of fruit extracts on liver histology, body weight and adipocyte in pre-pubertal female mice

**Figure 2:**
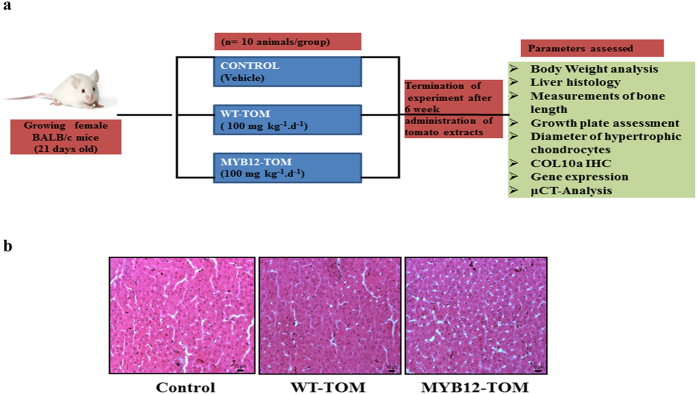
Animal experimental details and liver toxicity. (**a**) Experimental design for animal studies. (**b**) Representative sections of liver tissue of control, WT-TOM and MYB12-TOM with HE staining. Photographs are taken at 10X. The photograph was taken by DC.

**Figure 3:**
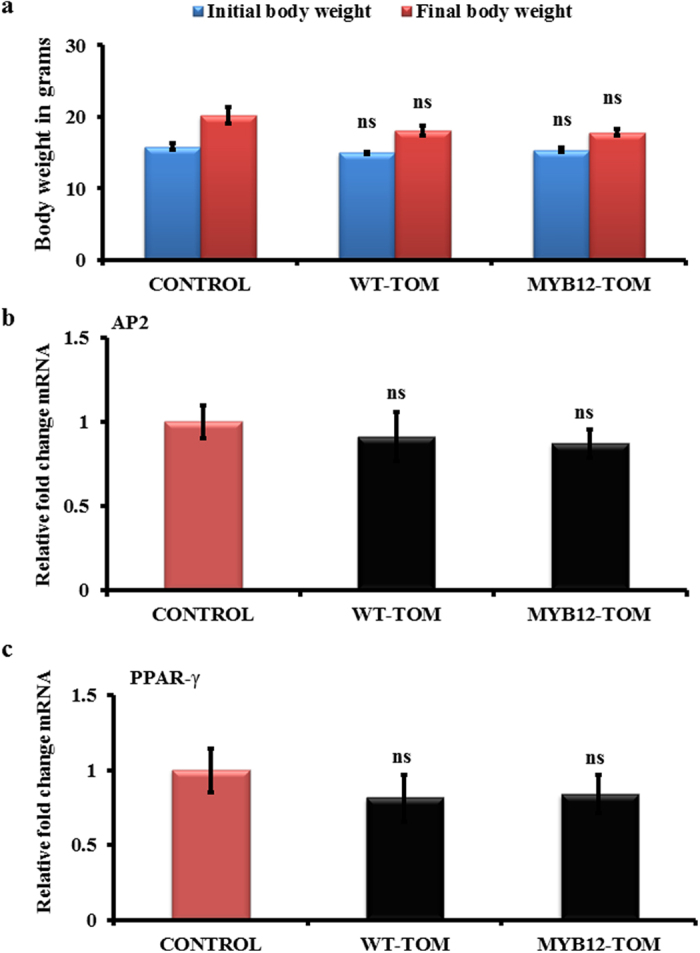
Effect of tomato fruit extract on body weight and adipogenic mRNA expression in growing mice. (**a**) Body weight changes during the treatment period. (**b,c**) represents fold change in mRNA levels of AP2 and PPARγ respectively after normalizing with GAPDH mRNA levels (n = 5 in each group). All values are expressed as mean ± SEM mice/group ns = not significant.

To study the effect of enhanced flavonoid content in transgenic fruits in animal system, extracts from MYB12-TOM and WT-TOM were administered (100 mg kg^−1^.d^−1^) to growing female BALB/c mice daily for 6 weeks and different parameters were analysed (Fig. 2a). For safety assessment of the extracts of WT-TOM and MYB12-TOM, mice were analysed for liver toxicity by histological investigation of the hepatocytes. Hepatic inflammation was not observed after MYB12-TOM and WT-TOM tomato extract consumption. Normal sinusoidal Kupffer cells in both vehicle and tomato extracts treated animals were observed without any morphological variations suggesting that tomato extracts were safe for administration in animals (Fig. 2b). MYB12-TOM and WT-TOM fruit extracts, after six week consumption, resulted in no significant differences in the initial and final body weight when compared with controls (mice receiving vehicle) (Fig. 3a). Data also shows no significant change in expression of adipogenic genes like, adipocyte fatty acid binding protein (AP2) and peroxisome proliferator activated receptor gamma (PPARγ) even after six weeks administration of the extract (Fig. 3b,c).

### Effect of MYB12-TOM on bone length parameter in female BALB/c mice

**Figure 4:**
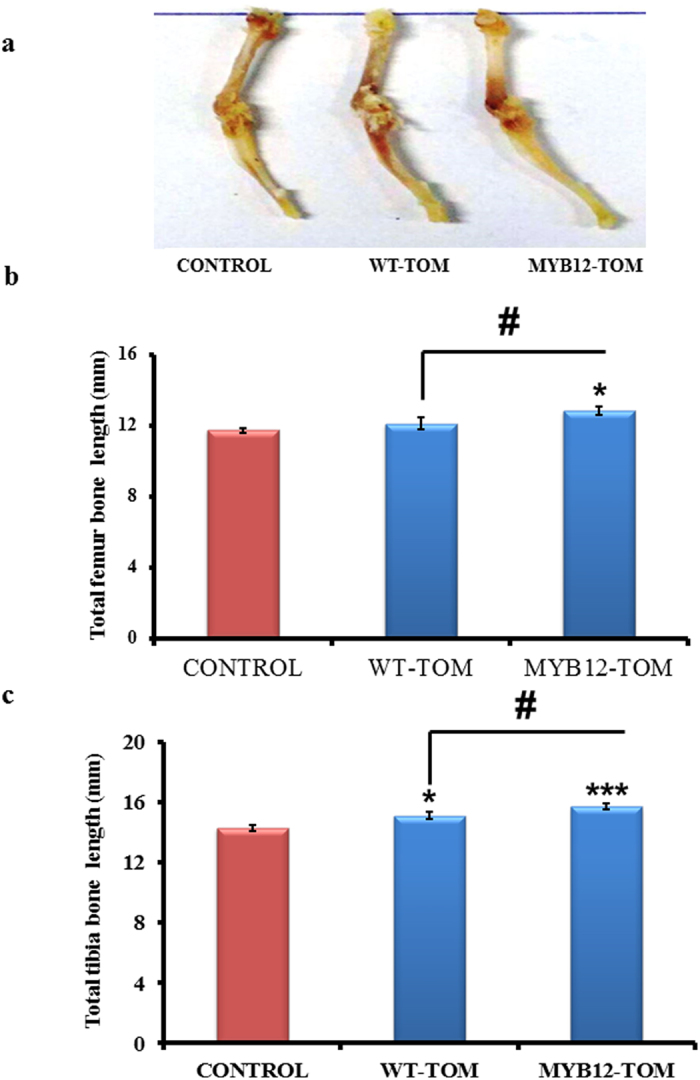
Effect of tomato fruit extract on femur and tibia height. (**a**) Representative images of femur and tibia after administration of tomato extracts from different groups. (**b,c**) length in the femur and tibia respectively as measured by digital calipers. All values are expressed as Mean ± SEM (n = 10) mice/group. *P < 0.05, ***P < 0.001 compare to control and #P < 0.05 compared to WT-TOM group. The photograph was taken by DC.

The proliferation of the chondrocytes is an important phenomenon for longitudinal bone growth. At the end of experiment, tibia as well as femur length was measured from proximal to distal ends. The growth analysis indicates that MYB12-TOM significantly increased femoral and tibial length compared with WT-TOM and vehicle control group animals (Fig. 4). Digital caliper measurements suggests that femurs of the mice groups treated with extracts from WT-TOM and MYB12-TOM fruits increased approximately 3.13% and 9.4% (P < 0.05) in length respectively as compared to control group. Extracts from MYB12-TOM increased the femur length significantly by 6.1% (P < 0.05) as compared to WT-TOM. In case of tibia, WT-TOM and MYB12-TOM increased the bone length by 5.54% (P < 0.05) and 9.98% (P < 0.001) as compared to control group respectively. Further, comparisons within experimental groups show that MYB12-TOM increased tibial length significantly by 4.2% (P < 0.05) as compared to WT-TOM group (Fig. 4b,c).

**Figure 5:**
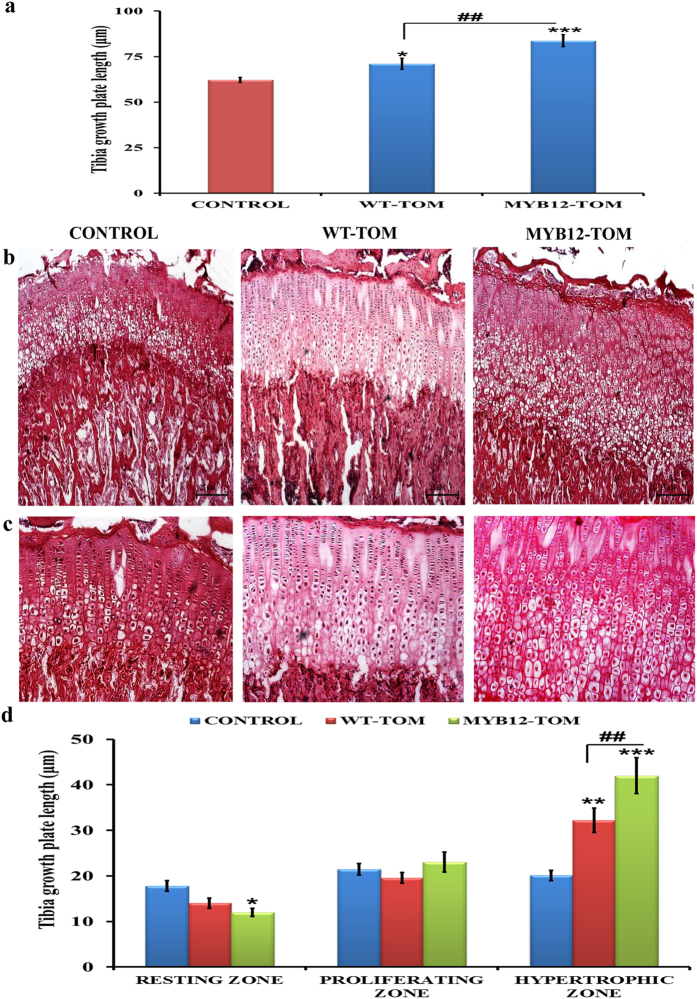
Effect of tomato fruit extract on tibial height and growth plate zones. (**a**) Measurement of tibial growth plate height in CONTROL, WT-TOM and MYB12-TOM animal groups. Panels (**b,c**) are representative images of growth plate zones of tibia of different experimental groups. (**d**) Represents measurement of tibial growth plate height of different zones i.e resting, proliferating and hypertrophic zones. All values are expressed as mean ± SEM (n = 10 mice/group). *P < 0.05, **P < 0.01, ***P < 0.001 compared to control and ^##^P < 0.01 compare to WT-TOM group.

To further identify the underlying mechanism behind the above observed effects, histomorphometric analysis of the proximal tibial epiphysis growth plates was performed after 6 weeks of treatment. We measured the height of the growth plate region (Fig. 5a). Our observations suggest that in MYB12-TOM group, height increased by 34.78% (P < 0.001) and 17.88% (P < 0.01) as compared to control (vehicle) and WT-TOM group respectively. The growth plate consists of four distinctive histological zones beginning from resting zone and extending through proliferative, hypertrophic and ossification zone. In the ossification zone, the chondrocytes eventually die and get transformed into bone matrix where the longitudinal bone growth occurs^18^. In our study, histological and cellular changes in the growth plate with minimal distortion in cellular columination and morphology was observed by haematoxylin and eosin staining (Fig. 5b–d). Sections of proximal tibia revealed significant changes in the growth plate in MYB12-TOM group animals as compared to other two groups. Histomorphometric measurements corroborated the above data showing that treatment with WT-TOM and MYB12-TOM increased the total growth plate and bone height of both tibia and femur that resulted in the total increase in the bone length. WT-TOM and MYB12-TOM fruit extracts had the potential to increase the height and width of hypertrophic zone, the cells that finally mineralize to form bone. However, this enhancement was significantly higher in case of mice administered with MYB12-TOM fruit extract as compared to WT-TOM fruit extract. The height of the hypertrophic region in growth plate of pre-pubertal female mice increased significantly in WT-TOM (P < 0.05) and MYB12-TOM (P < 0.001) compared to control group. In MYB12-TOM treated mice, significantly increased (P < 0.01) hypertrophic zone height was observed compared to WT-TOM animal group after treatment of 6 weeks (Fig. 5e).

### Effect of COL10a expression in the hypertrophic region of growth-plate

**Figure 6:**
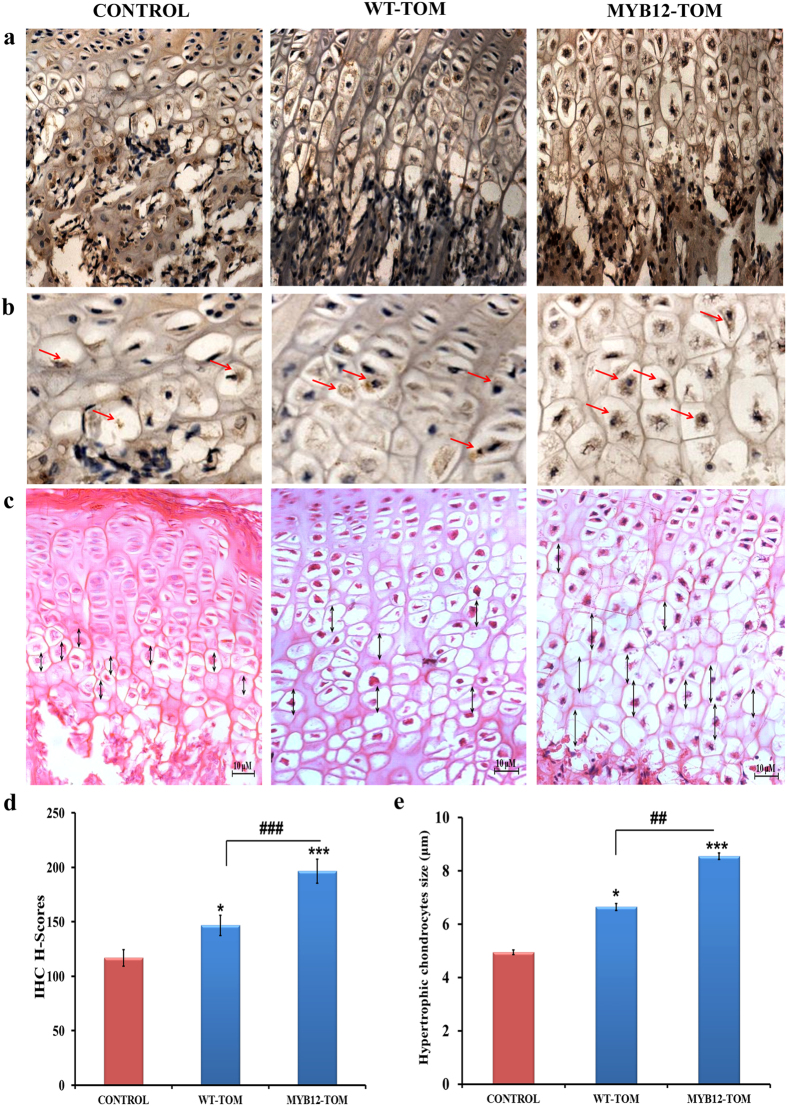
Immunostaining of chondrocytes with COL10A and hypertrophic cell size measurement of chondrocytes in the growth plate region of tibia. (**a,b**) Representative images of immunostaining with COL10A in growth plate of tibia in different groups. (**c**) Represents H&E staining to show hypertrophic cell size in the growth plate region of control, WT-TOM and MYB12-TOM groups. (**d**) Represents COL10A positive cells with IHC H-score of the hypertrophic cells of growth plate. Ten images of hypertrophic regions of different samples of each group were taken, the cells were then marked and analyzed according to the H-score (**d**) Shows the quantification of the hypertrophic chondrocytes size that were marked parallel to the axis of the bone growth. Images were taken in 10 fields of tibia that were chosen randomly at hypertrophic region of bone. Data represents Mean ± SEM (n = 10) mice/group. *P < 0.05, ***P < 0.001 compare to control and ^##^P < 0.01, ^###^P < 0.001 compare to WT-TOM group.

COL10a immuno-histochemistry revealed considerable differences in the hypertrophic zone of different groups. COL10a is expressed in the hypertrophic zone and continues till the beginning of the calcification of the growth plate (Fig. 6a). In the control group, the characteristic longitudinal columnar pattern of the growth plate was maintained. However, the level of expression of COL10a was lower in control and WT-TOM than that of the transgenic MYB12-TOM group. Expression of COL10a protein were up-regulated (H-score ≥200) in MYB12-TOM treated tissues as shown in Fig. 6a,b. The H-scores of COL10a positive hypertrophic chondrocytes were 117, 146.8 and 206.5 in control, WT-TOM and MYB12-TOM respectively. The result shows that transgenic MYB12-TOM group exhibited significantly higher COL10a expression from control group (P < 0.001) and WT-TOM (P < 0.001) (Fig. 6d).

### Effect of MYB12-TOM on hypertrophic cell size

After H&E staining histological analysis showed increased hypertrophic cell size in the growth plate region after 6 week of administration of tomato extracts. Administration of MYB12 -TOM extract increased hypertrophic cell size by 72.7% (P < 0.001) compared to control group and 28.57% (P < 0.01) compared to WT-TOM (Fig. 6e). Markings as double headlines indicate the size of hypertrophic chondrocytes cells parallel to the axis of bone growth in the growth plate region (Fig. 6c). Thus, it is apparent that MYB12-TOM is responsible for the interstitial growth of the epiphyseal plate which, in turn, determined the rate of bone elongation or longitudinal growth.

### Effect of MYB12-TOM on gene expression

**Figure 7:**
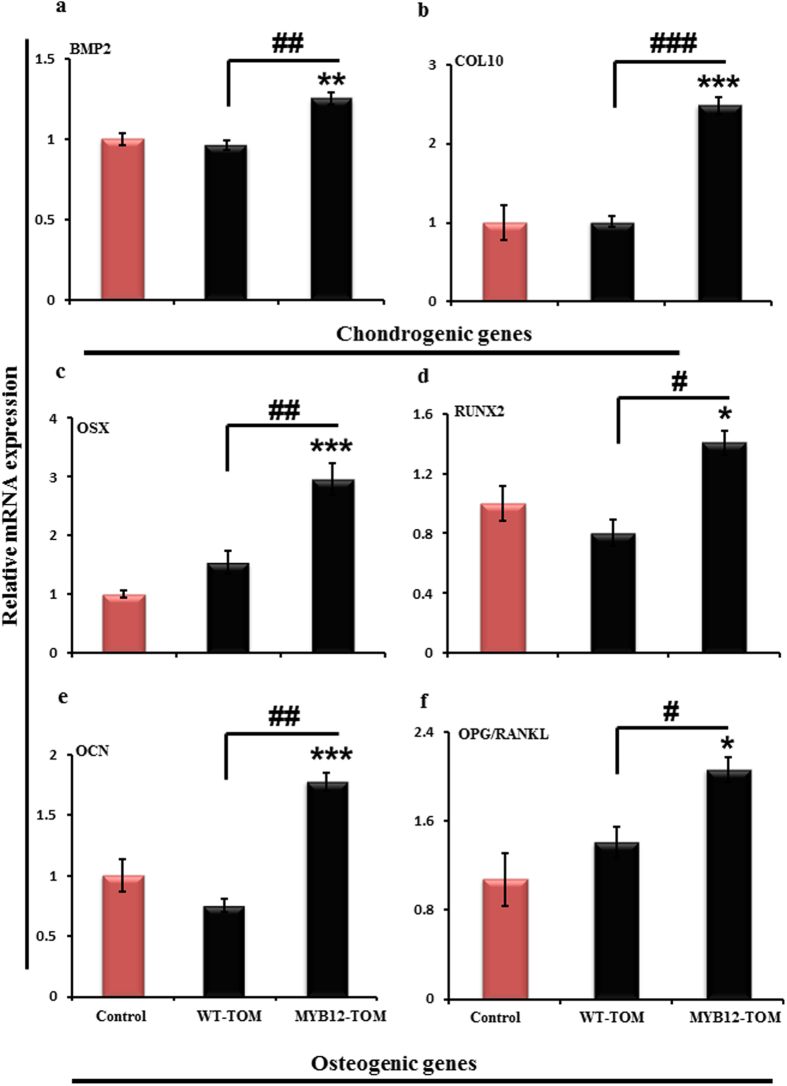
Effect of tomato fruit extract on chondrogenic and osteogenic mRNA expression in growing mice. (**a,b**) represent fold change in mRNA levels of BMP2 and COL10A after normalizing with GAPDH mRNA levels (n = 5 in each group). (**c**) OSX (**d**) RUNX2 (**e**) OCN and (**f**) OPG/RANKL ratio fold change in mRNA levels after normalizing with GAPDH mRNA levels. All values are expressed as mean ± SEM (n = 5 mice/group). *P < 0.05, **P < 0.01, ***P < 0.001 compare to control and ^#^P < 0.05, ^##^P < 0.01, ^###^P < 0.001 compare to WT-TOM group.

Gene expression analysis at the site of growth plate in MYB12-TOM group of animals shows significantly increased expression of BMP2 compared with WT-TOM and control group (Fig. 7a). Further, we observed that the expression of COL10, marker for hypertrophic cells, also significantly increased (by ~2.5 fold) suggesting greater proliferation of cells after MYB12-TOM administration as compared to WT-TOM and control group of animals (Fig. 7b). These results indicate that growth plate cartilage chondrocytes and metaphyseal bone cells respond to MYB12-TOM fruit extracts treatment and have protective effects on metaphyseal bone and the potential to increase the bone length.

Assessment of relative gene expression in the region below the growth plate suggests enhanced expression of osteogenic genes, Osterix (OSX), Runt related transcription factor (RUNX2) and osteocalcin (OCN) in MYB12-TOM group of animals as compared to other groups (Fig. 7c–e). RUNX2 and OSX are essential for osteoblast differentiation and bone morphogenesis while OCN (bone gamma-carboxyglutamic acid containing protein) is used as marker for bone formation process and is implicated in mineralization of bone. RUNX2, Osteocalcin and Osterix increased by 1.5 (P < 0.05), 2 (P < 0.001), and 3 (P < 0.001) fold, respectively in MYB12**-TOM group as compared to control group. Significant increase (P < 0.05) in the overall OPG/RANKL ratio, indicator of bone formation rather than bone resorption, was also observed in MYB12-TOM treatment group (Fig. 7f).

### Effect of MYB12-TOM on trabecular micro-architecture of long bones

**Figure 8:**
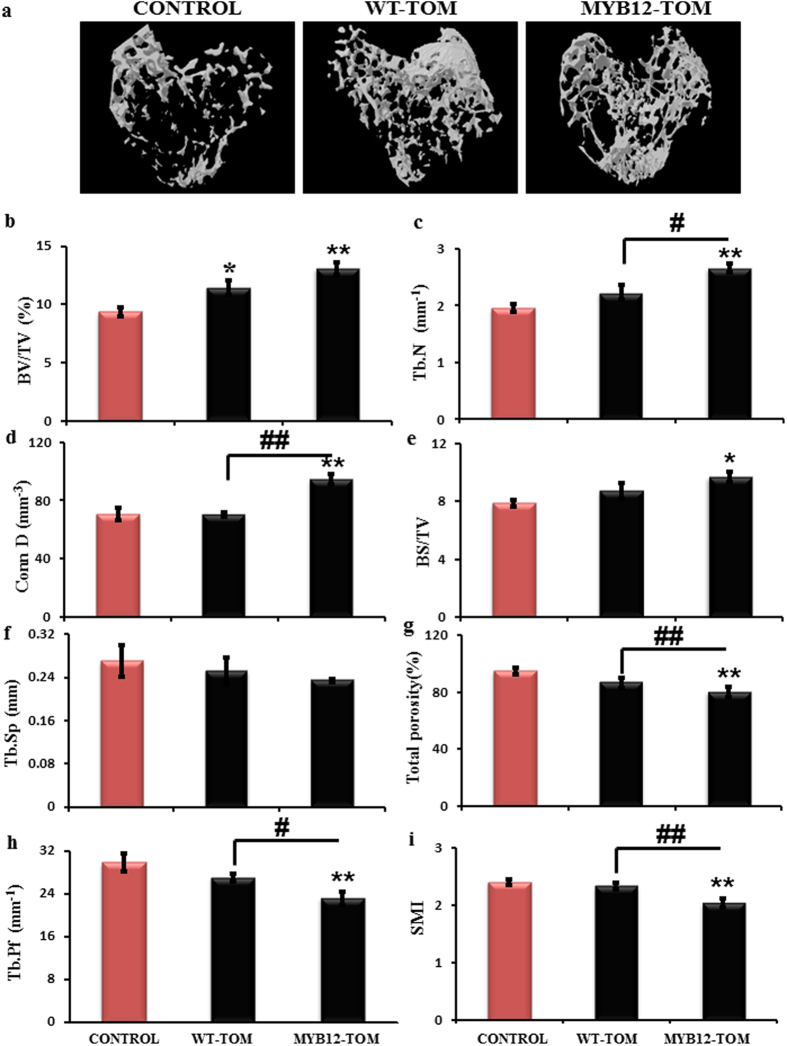
μCT analysis of various trabecular parameters of tibia. **(a)** Representative images depicting 3-D structure of tibia bone from animals control and animals treated with WT-TOM and MYB12-TOM extracts. The trabecular parameters including (**b**) BV/TV, (**c**) Tb.N, (**d**) Conn D, (**e**) BS/TV, (**f**) Tb.Sp, (**g**) Total Porosity, (**h**) Tb.Pf, (**i**) SMI. All the values are expressed as mean ± SEM (n = 6 mice/group). *P < 0.05, **P < 0.01, ***P < 0.001 compare to control and ^#^P < 0.05, ^##^P < 0.01, ^###^P < 0.001 compare to WT-TOM group.

At the end of the treatment period, trabecular architecture of isolated femur and tibia in all the groups was studied using 3D-μCT. μCT changes were observed in the tibial metaphyseal region, trabecular structural changes were clearly visible in control, WT-TOM and MYB12-TOM groups as represented in Fig. 8a. For Quantitative parameters, analysis was carried out for BV/TV (Fig. 8b) that indicates the fraction of a given volume of interest (the Total Volume TV) that is occupied by mineralized bone (Bone Volume). Results suggest that MYB12-TOM improved the bone quality by increasing BV/TV up to 39.9% (P < 0.01), as compared to control group. This was followed by increase in trabecular number (Tb.N), (Fig. 8c) which shows dense collagenous tissue leading to increase in the connection density (Conn.D) (Fig. 8d), surface area of trabeculae (BS/TV) (Fig. 8e) and reduction in the total porosity in bone tissue. MYB12-TOM significantly increased the trabecular number (Tb.N) by 35.7% (P < 0.01) connective density (Conn.D) by 33.96% (P < 0.01) bone surface/tissue volume (BS/TV) by 22.99% (P < 0.05) compared to control. MYB12-TOM administration decreased trabecular separation (Tb.Sp) (Fig. 8f) by 11.11%. MYB12-TOM treatment significantly decreased total porosity to 15.59%, P < 0.01 (Fig. 8g) and trabecular bone pattern factor Tb.Pf to 22.56%, P < 0.01 (Fig. 8h) this reflects greater trabecular connectedness with MYB12 TOM administration, such that greater the connectivity, decreased is the the Tb.Pf and total porosity as compared to the control group. Finally, data of Structure Model Index (SMI) (Fig. 8i) that is an indirect indicator of bone strength was estimated. MYB12-TOM treatment significantly decreased the SMI values by 12.0% (P < 0.01) compared to control. When MYB12-TOM compared to WT-TOM, MYB12-TOM significantly increased Tb.N (P < 0.05), Conn.D (P < 0.01) and decreased total porosity (P < 0.01), Tb.Pf (P < 0.05), SMI (P < 0.01). The similar pattern of changes in bone parameters were observed in the femur region (Supplementary Table 1). Overall, data suggests that 6 weeks of tomato extract supplementation results in significantly increased bone volume in MYB12-TOM extract compared to control or WT-TOM fruit extract.

## Discussion

Height and growth rates are important indicators of health in infants and adolescents. Much more bone is deposited by the process of modeling versus remodeling as the skeleton grows in both size and density in children and adolescents. Most people of short stature do not have a pathological condition per se but merely exhibit some deviations from the normal parameters of growth. Our previous studies using phytopharmacological approach have shown beneficial effects for bone health^19^. Although certain plants are rich sources of flavonoids, yet many commonly consumed food remain deficient in their contents. For obvious reasons, genetic modification of flavonoid biosynthesis in crop plants is desirable for developing dietary solutions for flavonoids.

Flavonoid biosynthesis has been manipulated in different plant species through homologous or heterologous expression of regulatory and structural genes of flavonoid pathway^17^,^20^,^21^. Despite considerable research focused on genetic manipulation of flavonoid biosynthesis in plants having been conducted, there are only limited reports evaluating the health benefits of genetically engineered plants^14^. Earlier, transgenic tomato fruits enriched with anthocyanin were reported to exhibit anti-carcinogenic effects. In addition, enhanced accumulation of certain flavonoids has been demonstrated to improve bone health in different animals^13^. Tomato fruits are highly deficient in flavonoids owing to the limited expression of genes involved in flavonoid biosynthesis. However, its enormous consumption across the biosphere makes it a lucrative fruit for genetic modification. AtMYB12, an activator transcription factor, activates the expression of complete set of genes committed to flavonoid biosynthesis and thereby overall biosynthesis^19^. In our previous study, constitutive expression of AtMYB12 transcription factor in tomato led to altered genome-wide expression in fruit and leaf with significant enhanced accumulation of flavonols such as rutin, quercetin and kaempferol and chrologenic acid as compared to non-transgenic plants^14^.

Therefore in this study, we explored the effect of nutritionally rich transgenic tomatoes for longitudinal height attainment in growing female rodents. Contrary to males, in females the attainment of peak bone mass is restricted only to the pre-pubertal stage. When there is commencement of estrogen hormone at the time of puberty, it marks the reduce in longitudinal bone growth^22^,^23^,^24^,^25^. The goal of this study was to assess increase in bone length accomplishment that will not only support increase in height but also improved bone mass reservoir before puberty by oral administration of MYB12–TOM extract. We observed overall increase in bone length in MYB12–TOM treatment group over a period of six weeks. The bones of MYB12-TOM group were significantly longer (Fig. 4b,c) with enhanced bone volume as depicted by decrease in trabecular separation parameter quantitated by μCT (Fig. 8). Decrease in Tb.Sp with increase in number of trabeculae in tibia and femur improved bone quality corroborated with the increased connective density and decreased porosity of bone. Overall this improved the bone microarchitecture. Gene expression of the osteogenic and chondrogenic specific genes supports the enhanced bone formation especially at the site of growth plate of growing bones in MYB-TOM group of animals as compared to other groups. In addition, increased OPG/RANKL ratio indicates that the extract from transgenic fruit with enhanced flavonol content favors bone osteoblasts formation as is evident from increased BMP2, RUNX2 and OSTEOCALCIN expression and increase bone anabolic effect by acting on OPG/RANKL ratio (Fig. 7).

The epiphyseal plate region is mainly responsible for the elongation of the long bones. The bone elongation function is carried out by interstitial expansion of chondrocyte cells while one end is continuously replaced by a calcified matrix to convert to bone. This interstitial expansion happens through a combination of chondrocyte proliferation and enlargement of the lacunae that surround these cells. Our results show that MYB12-TOM treatment increased the expression of COL10 indicating increase in number of hypertrophied cells which in turn contributed to the expansion of the lacunae in the growth plate. From this data, it is apparent that MYB12-TOM may be responsible for the interstitial growth of the epiphyseal plate which, in turn, determined the rate of bone elongation (Fig. 6).

Peak Bone Mass (PBM) is influenced by a variety of factors including nutrition and physical activity which can improve bone health^26^,^27^ with studies suggesting that natural sources of flavonoids being good for bone health with improved bone microarchitecture and helps in increase in longitudinal bone length^8^,^9^,^28^.

Through this study, we demonstrate that development of nutritionally-rich transgenic food can positively affect growing animals. We observed that the transgenic fruit extract rich in flavonoids affected cellular processes at the growth plate, metaphyseal bone, and bone marrow which directly impacted bone lengthening, bone mass accumulation, and modulation of cells towards osteoblastic lineage (Fig. 6a,b). It is apparent from this study that the transgenic fruit extract from MYB12-TOM tomatoes significantly increased the height of bones by increasing the size and number of hypertrophic cells (Figs 5a,d and 6). This is corroborated with the increased gene expression of BMP2 and COL10a mRNA in the chondrogenic region of tissue. Increased expression of BMP2 results in higher number of chondrogenic cells, thus further increasing the bone length^29^,^30^.

In conclusion we demonstrate the effect of naturally occurring phytoestrogens on growing rodents through pathway engineering of flavonoid biosynthesis. This will address the problem of bone growth defects such as short height and delay in peak bone mass achievement in females. In the present study performed in pre-pubertal female mice, we show that MYB12-TOM significantly improves bone growth by increasing the height of growth plate via increased number and size of hypertrophic chondrocytes that promotes longitudinal growth with markedly improved bone microarchitecture.

## Methods

### Plant material and phytochemical analysis

#### Solanum lycopersicum var

Pusa early dwarf (tomato) WT as well as AtMYB12 expressing transgenic lines have been used in this study^17^. Tomato plants were grown in glass house at 22 °C ± 2 °C and 16hr/8hr light-dark photoperiods. Fruit samples were harvested at red ripe stage and frozen in liquid nitrogen and kept in −80 °C deep freezer until further use. Extraction and analysis of flavonoid through HPLC and antioxidant activity was carried out essentially as previously described^17^. For animal experiments, ethanolic extract was prepared and used as per method descried in Pandey *et al*., (2014)^13^. The experiment was performed by using three independent replicates.

### Animal experiment

All experiments were performed in accordance with IAEC (Institutional Animal Ethical Committee, New Delhi, Ref. No. IEAC/2013/19) guidelines. Experimental protocols were approved by CSIR-Central Drug Research Institute, Lucknow. We obtained 21 days old female BALB/c mice weighing 12–15 gm each from animal house. All animals were individually housed at 22-24 °C in 12hr/12hr light-dark cycles. Normal chow diet and water were provided *ad libitum*. After acclimation for 48 hr, 10 mice were randomly assigned to three groups. The proposed study was performed to compare the *in vivo* effect of the continuous administration of CONTROL and WT-TOM (tomato fruit crude extracts) versus MYB12-TOM (transgenic tomato standardized fruit crude extracts) (100 mg kg^−1^ d^−1^) for a period of 6 weeks.

### *In vivo* toxicity and liver histology

Following treatment with WT-TOM and MYB12-TOM, liver tissue from different groups were collected and fixed in 4% paraformaldehyde. Sample were dehydrated in ascending grades of isopropanol, cleared in xylene and embedded in paraffin wax using standard procedures. Transverse sections of 5 μm were stained with haematoxylin and eosin and representative images were captured using Nikon Eclipse 80i^31^.

### Micro–computed topographic (μCT) analysis

Micro–computed topographic (μCT, 3D) determination of excised bones was carried out using the Sky Scan 1076 μCT scanner (Sky Scan, Ltd.Q16, Aartselaar, Belgium) as described in previously published reports^31^,^32^,^33^,^34^. Femurs and tibias were dissected from the animals after euthanasia, cleaned of soft tissue, and fixed before storage in alcohol. The samples were scanned in batches of three at a nominal resolution (pixels) of 9 micron. Reconstruction was carried out using a modified Feldkamp algorithm using the Sky Scan Nrecon software, which facilitates network-distributed reconstruction carried out on computers running simultaneously. The X-ray source was set at 50 kV and 200 mA, with a pixel size of 9 μm. Three-dimensional reconstruction of bone was performed using the triangulation algorithm. Both femoral and tibial trabecular region were selected with reference to the growth plate. In brief, the cross sectional slice is selected as a growth plate reference slice followed by moving slice-by-slice toward the growth plate from the metaphysis/diaphysis. Through this, a point is reached where a clear “bridge” of low density cartilage (chondrocyte seam) becomes established from one corner of the cross section to another. This bridge is established by the disappearance of the last band of fine primary spongiosal bone interrupting the chondrocyte seam. This landmark allows a reference level to be defined for the growth plate: trabecular volumes of interest are then defined relative to this reference level. Therefore, even though the bone length differs in different groups but the reference region for micro-CT analysis remains the same starting from primary spongiosa (terminal region of hypertrophic zone). This landmark allows a reference level to be defined for the growth plate: trabecular volumes of interest are then defined relative to this reference level and its region was equal for all groups. The trabecular BV/TV (%), trabecular number (Tb.N) (mm^−1^), trabecular connection density (mm^−3^), and trabecular separation (Tb.Sp) (mm), trabecular porosity were directly measured on three-dimensional images. The trabecular bone pattern factors (Tb.pf) (mm^−1^) and the structure model index were computed using software provided with the μCT machine.

### Reverse transcriptase polymerase chain reaction (RT-PCR)

The bones were excised during autopsy, cleaned and collected in RNA later. For RNA isolation, bones were cut in two parts according to one upper head part with growth plate region for chondrogenic genes and below growth plate portion for osteo- and adipogenic genes expression study. We marked the bones and cut them by fine bone cutter blade (Leica, USA). After separation the bones were separately crushed in liquid nitrogen and RNA was isolated by trizol method for qPCR experiments. cDNA was synthesized with a RevertAid cDNA synthesis kit (Thermo Scientific, USA), using 2 μg of total RNA. SYBR green (PureGene, USA) chemistry was used to perform the quantitative determination of relative expression of all genes. Real Time PCR (qPCR) was performed to assess the expression of osteoblast, adipocytes, and chondrocyte specific genes from femur and tibia of mice from our previously published protocol^35^,^36^,^37^. The house-keeping gene GAPDH was used as the internal control in this study. Primers were designed using the Universal Probe library (Roche Applied Sciences, USA) for different genes (Supplementary Table 2). These genes were analyzed using the Light Cycler 480 (Roche Molecular Biochemical’s, USA) real time PCR machine^35^,^36^,^37^,^38^.

### Histomorphometric analysis of growth plate

During the time of autopsy, femur and tibia were dissected out, cleaned and decalcified in 2% EDTA. For morphometric analysis of the growth plate region, three sections of 5 µm size of bone were obtained from tibia after decalcification and were mounted on a glass slide. Paraffin sections were de-waxed re-hydrated and were stained with haematoxylin and eosin (H&E). The total width of the growth plate at the proximal end of each tibia was measured along longitudinal axis oriented 90° to the transverse plane of the growth plate. At least ten measurements were obtained from each epiphyseal growth plate and final width determination in individual animals indicated the average of these values^38^,^39^. Regions along the centre of the bone were selected for measurements of the growth plate height. Heights were measured beginning from the resting zone till bottom of the section where calcification begins (end of growth plate region). Zones were demarcated according to the morphology of the cell types. In the resting zone, the cells lie within the reserve zone and can be identified as small, uniform, compactly and singly located chondrocytes. Proliferative cells were marked as flat and well divided in to longitudinal columns with mitotic activity at base in this zone. Below the proliferative zone, hypertrophic cells were characterized with no cellular division which finally differentiate terminally as swollen and then degeneration^40^,^41^. Height of different growth plate zones was measured using an image analysis system with the Image Pro Plus software (Media Cybernetics, Silver Springs, MD). Sections were viewed by light microscopy using a bright field illumination (Nikon Eclipse 80i) and images were captured in computer. The average zonal heights of the growth plate cartilage were obtained by measuring heights of the resting, proliferative and hypertrophic zones of the entire growth plate. Size of specific hypertrophic chondrocytes cells were measured parallel to the axis of the bone growth in the growth plate region.

### Immunohistochemistry of COL10a in the growth plate region of tibia

Mice treated with CONTROL, WT-TOM and MYB12-TOM groups were used for immunohistochemistry of COL10a. Immunohistochemistry was performed as per previously published protocol^42^ with slight modifications. Bone samples of different groups were rinsed under running water and stored in 70% ethanol for a minimum of 72 hr after decalcification. Tissue dehydration and processing were done as per standard protocols. The bones were then embedded in paraffin and 5 μm longitudinal sections were cut using microtome (LEICA USA microtome). Sections were dried at 37 °C in the oven for 4 hr before tissue processing for immunohistochemistry. Sections were deparaffinized and rehydrated in PBS, then pre-treated for antigen retrieval in a 10 mM citrate buffer for 10 min in a water bath heated to 55–60 °C. Immunohistochemistry was done using Vectastain ABC kit and procedure was according to the manufacturer (PK-4001, Vector Laboratories, CA, USA). Tissue sections were blocked in 5.0% BSA for 1 h and subsequently incubated with Rabbit polyclonal antibody COL10a1 (Merck Millipore 234196) (1:100) dilution in PBS containing 2.0% BSA (Sigma) at 4 °C in a humidified chamber for 4 h. Tissue sections were incubated with biotinylated secondary antibody and later, with ABC reagent for 1 h. Color was developed with the help of 3, 3′-diaminobenzidine (DAB) peroxidase substrate (SK-4100, Vector Laboratories, CA, USA) for 1 min and counter-stained with hematoxylin (51275, Sigma-Aldrich Inc., MO, USA)

All of the COL10a immuno-stained sections were reviewed and scored independently in a blinded manner, based on the H-score method, which reflects the staining intensity collected with the percentage of cells staining positively. For H-score method, 10 fields were chosen randomly at 40× magnification. The staining intensity in the cells was scored as 0, 1, 2 and 3 corresponding to the zero, weak, intermediate and strong staining, respectively. In each field, the total number of cells as well as cells stained at each intensity was calculated. The H-score was calculated following the formula: (% of cells stained at intensity category 1 × 1) + (% of cells stained at intensity category 2 × 2) + (% of cells stained at intensity category 3 × 3). H-scores varied from 0 to 300 where 300 represented 100% of cells strongly stained (3+)^43^,^44^.

### Statistical analysis

Data are expressed as mean ± SEM unless otherwise indicated. The data obtained in experiments with multiple treatments were subjected to one-way ANOVA followed by post hoc Newman-Keuls multiple comparison test of significance using GraphPad Prism 5.0 software. Qualitative observations have been represented following assessments made by three individuals blinded to the experimental designs.

## Additional Information

**How to cite this article**: Choudhary, D. *et al*. Genetically engineered flavonol enriched tomato fruit modulates chondrogenesis to increase bone length in growing animals. *Sci. Rep*. **6**, 21668; doi: 10.1038/srep21668 (2016).

## Supplementary Material

Supplementary Information
