# Supplementary material for: Genetically engineered flavonol enriched tomato fruit modulates chondrogenesis to increase bone length in growing animals
Source: Sci Rep. 2016 Feb 26;6:21668. doi: 10.1038/srep21668 (PMC4768317; doi:10.1038/srep21668)
Supplement: Supplementary Information [file srep21668-s1.pdf]

Genetically engineered flavonol enriched tomato fruit modulates chondrogenesis to increase bone length in growing animals

Dharmendra Choudhary[1,$], Ashutosh Pandey[2,$], Sulekha Adhikary[1], Naseer Ahmad[1], Chitra Bhatia[2], Sweta Bhambhani[2], Prabodh Kumar Trivedi[2,*], Ritu Trivedi[1,*]

[1]CSIR-Central Drug Research Institute (CSIR-CDRI), Endocrinology Division, Jankipuram Extension, Sitapur Road, Lucknow-226021, INDIA

[2]Council of Scientific and Industrial Research-National Botanical Research Institute (CSIR-NBRI), Rana Pratap Marg, Lucknow-226 001, INDIA.

[$]Present address (AP): National Agri-Food Biotechnology Institute (NABI), Mohali-160071, Punjab, INDIA

$: Contributed equally to this study

*Authors for correspondence

RT: ritu_trivedi@cdri.res.in, ritu_pgi@yahoo.com

PKT: prabodht@hotmail.com, prabodht@nbri.res.in

Running title: Nutritionally rich transgenic tomato fruit for increased bone length in mice

Supplementary Table 1: Femur microstructure parameters after 6 week treatment

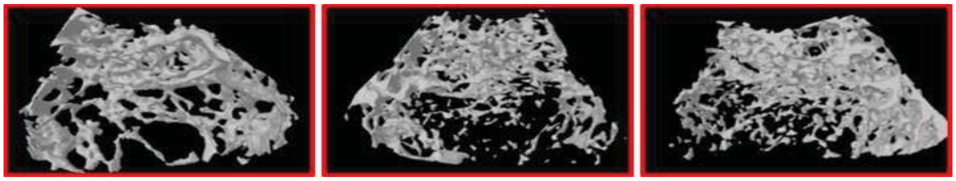

Microarchitectural parameters	CONTROL	WT-TOM	MYB12-TOM
BV/TV (%)	13.32±0.66	15.9±1.33	18.72±.54 **
Tb.N (mm^{-1})	2.52± 0.08	2.71± 0.08	3.13± 0.17**#
Conn D (mm^{-3})	153± 9.63	165.4± 11.32	202.6± 3.84 **#
BS/TV	9.6±0.3	9.92±0.95	12.35±0.55*
Tb.Sp (mm)	0.24±0.01	0.23±0.01	0.19±0.01*
Total porosity (%)	90.9±3.65	85.52±2.55	79.16±2.78*
Tb.Pf (mm^{-1})	15.8±0.66	13.06±0.95*	11.44±0.54**
SMI	1.96±0.10	1.69±0.06*	1.58±0.04**

BV/TV, bone volume fraction; Tb.N, trabecular number; Conn.Dn, connectivity density; BS/TV Bone surface density; Total porosity (Po) (%); Tb.Pf ,Trabecular pattern factor; Tb.Sp, trabecular separation; SMI, structure model index.

$^{**}P < 0.01$, $^{*}P < 0.05$, when compared with the control group,

$^{\#}P < 0.05$ when compared with the WT-TOM group

Supplementary Table 2 : List of the primers used in the present study

Oligo name	Sequence (5’ to 3’)	Purpose
OCN Far	TGAGGACCATCTTCTGCTCA	For Real time PCR
OCN Rev	TGGACATGAAGGCTTTGTCA	For Real time PCR
PPAR-γ Far	GAAAGACAACGGACAAATCACC	For Real time PCR
PPAR-γ Rev	GGGGGTGATATGTTTGAACTTG	For Real time PCR
OPG Far	AGCCATTGCACACCTCAC	For Real time PCR
OPG Rev	CGTGGTACCAAGAGGACAGAGT	For Real time PCR
RANKL Far	AGCCATTTGCACACCTCAC	For Real time PCR
RANKL Rev	CGTGGTACCAAGAGGACAGAGT	For Real time PCR
GAPDH Far	AGCTTGTCATCAACGGGAAG	For Real time PCR
GAPDH Rev	TTTGATGTTAGTGGGGTCTCG	For Real time PCR
BMP2 Far	AGATCTGTACCGCAGGCACT	For Real time PCR
BMP2 Rev	GTTCCTCCACGGCTTCTTC	For Real time PCR
Col10a1 Far	GCCCAGCTGGAATTTCTGT	For Real time PCR
Col10a1 Rev	CCTGGGGCACCTGTAAGTC	For Real time PCR
aP-2(FABP4) Fa	GAAAACGAGATGGTGACAAGC	For Real time PCR
aP-2(FABP4) Re	GCCCTTTCATAAACTCTTGTGG	For Real time PCR
OSX Far	TGCTTCCCAATCCTATTTGC	For Real time PCR
OSX Rev	AGCTCAGGGGGAATCGAG	For Real time PCR
